# Effects of *Helicobacter pylori* Infection on Ghrelin and Insulin-like Growth Factor 1 Secretion in Children with Idiopathic Short Stature

**DOI:** 10.3390/jcm11195868

**Published:** 2022-10-04

**Authors:** Marzena Kolasa-Kicińska, Renata Stawerska, Paweł Stawerski, Andrzej Kałużyński, Elżbieta Czkwianianc, Andrzej Lewiński

**Affiliations:** 1 Department of Endocrinology and Metabolic Diseases, Polish Mother’s Memorial Hospital–Research Institute, 93-338 Lodz, Poland; 2 Department of Paediatric Endocrinology, Medical University of Lodz, 93-338 Lodz, Poland; 3 Consilio Diagnostyka, Laboratory of Histopathology, 93-357 Lodz, Poland; 4 Department of Clinical Pathomorphology, Polish Mother’s Memorial Hospital–Research Institute, 93-338 Lodz, Poland; 5 Department of Gastroenterology, Allergology and Paediatrics, Polish Mother’s Memorial Hospital–Research Institute, 93-338 Lodz, Poland; 6 Department of Endocrinology and Metabolic Diseases, Medical University of Lodz, 93-338 Lodz, Poland

**Keywords:** idiopathic short stature, ghrelin, leptin, IGF-1, *Helicobacter pylori*, children

## Abstract

Background: A diagnosis of "idiopathic short stature" (ISS) in a child means that the cause of the disease has not been established, although there are certainly some unknown factors that contributed to its occurrence. Ghrelin and leptin are important in controlling food intake; ghrelin is also a growth hormone (GH) stimulator. Both enterohormones are produced in the stomach and their secretion may be affected by a *Helicobacter pylori* (*H. pylori*) infection. Methods: Our study included a group of 61 children (53 prepubertal and 8 peripubertal) with ISS, without any gastrointestinal tract symptoms but in whom the histopathological evaluation of stomach tissue was made during gastroscopy to diagnose *H. pylori* infection. In each child, fasting ghrelin, leptin and IGF-1 concentrations, and GH levels in two stimulation tests were assessed. Results: *H. pylori* infection was confirmed in 24.6% of the children. Ghrelin and IGF-1 concentrations were significantly lower in *H. pylori*-positive than *H. pylori*-negative children (this was more noticeable in prepubertal subgroups), however there was not a discrepancy in regards to GH concentrations in stimulation tests, leptin levels or the nutritional state between groups. Conclusions: Short children, infected by *H. pylori* seem to have lower ghrelin and IGF-1 concentrations than children without infection, this may be the reason for a worse growth rate in this subgroup.

## 1. Introduction

A child’s growth depends on the proper acting of growth hormone (GH) and its main mediator, insulin-like growth factor-1 (IGF-1), and the supply and absorption of the adequate amount of nutrients. In many short children, despite numerous tests, it is impossible to establish the cause of their inadequate height velocity and growth deficit. In such cases, idiopathic short stature (ISS) is diagnosed [1,2]; however, it can be assumed that many of these cases are due to undiagnosed abnormalities [1,2].

In our previous study, we detected as many as 70% of children initially diagnosed with ISS had various oligosymptomatic gastrointestinal diseases, including *H. pylori* infection, which was one of the most frequently observed disorders [3]. There are many reports and meta-analyses showing that *H. pylori* infection worsens the growth rate [4,5,6,7,8,9,10,11,12] and weight gain in children [13,14], but not all studies confirm these reports [15,16,17,18,19]. The diagnostic methods for detecting *H. pylori* infection vary between studies (including both non-invasive and invasive tests), while upper gastrointestinal endoscopy with biopsies for culture, histology and rapid urease test remains the “golden standard” [20]. Most of these studies report findings from the general pediatric population and only a few focus on the group of short stature children [21,22,23].

It should be emphasized that the prevalence of *H. pylori* infections worldwide is still rather high and ranges from 30% to 90% of the population [24,25]. It is estimated that in Poland it affects about 20–30% of children aged 2–17 [26]. The symptoms of *H. pylori* infection in children are relatively poorly expressed; slow height velocity may be one of them, however the reasons for the deceleration of height rate in *H. pylori*-positive children are unclear. In 2017, the European and North American Society of Gastroenterology and Nutrition presented their statement on the performance of diagnostic tests for *H. pylori* infection when examining the causes of short stature. They recommended against diagnostic testing for *H. pylori* in short stature children (recommendation 7), but the deceleration of linear growth should be treated as an alarm sign for testing *H. pylori* (recommendation 4) [20].

Two important hormones for children’s growth processes and weight gain are formed in the stomach. Ghrelin is produced by X/A cells in the gastric oxyntic mucosa, mainly in the gastric body and secreted into circulation [27,28,29]. Ghrelin, via its activating effect on the orexigenic centers, stimulates food intake, decreases energy expenditure and promotes weight gain, but it is simultaneously a hormone which stimulates the secretion of GH and, indirectly, the GH main mediator, IGF-1 [30]. Leptin is a peptide product of the OB gene, expressed mainly by adipocytes. Leptin stimulates the anorexigenic centers, reduces appetite and enhances thermogenesis and its levels in serum reflects body fat stores. However, it is also produced by endocrine P cells in the gastric mucosa and gastric leptin contributes to serum leptin levels [31]. Thus, diseases involving gastric mucosa can affect the production and secretion of both hormones. One of causes could be an *H. pylori* infection [32,33].

The issues of ghrelin and leptin concentration changes that result from the *H. pylori* infection are extremely interesting in regards to children with short stature. A reduced ghrelin concentration with a simultaneously increased leptin concentration in children may be associated with worse appetite. Since ghrelin is one of the strongest stimulants of GH secretion from the pituitary gland, its low concentration may reduce the spontaneous secretion of GH and IGF-1.

It was assumed that *H. pylori*-related chronic gastritis reduces the number of ghrelin immunopositive cells, suppresses ghrelin mRNA expression and decreases ghrelin concentration in serum [6,15], since more than 80% of ghrelin in blood serum originates from the stomach [33]. In turn, there is evidence that the *H. pylori* infection significantly increases gastric leptin expression, which is associated with the presence of multiple cytokines in the gastric inflammation process [33,34]. Moreover, it was reported that, following effective *H. pylori* eradication, ghrelin concentration rises, while leptin concentration falls and, additionally, appetite and body weight increase is observed [6,33,35,36]; however, there is very limited data suggesting growth rate improvement [6,13,35]. So far, there have been only a few publications concerning the influence of *H. pylori* infection on ghrelin and leptin concentrations in children with short stature [6,37].

The aim of this study was, therefore, to compare the concentration of ghrelin, GH, IGF-1 and leptin, as well as the nutritional status of children with ISS depending on the presence or absence of *H. pylori* infection, based on the histopathological evaluation of their gastric mucosa obtained from biopsies taken during upper gastrointestinal endoscopy.

## 2. Materials and Methods

### 2.1. Basic Data on the Method of Performed Measurements (Height, Weight, Growth Rate and Puberty)

The study included children admitted to the Department of Endocrinology and Metabolic Diseases at Polish Mother’s Memorial Hospital–Research Institute (PMMH-RI) in Lodz for two consecutive years, in order to conduct hormonal diagnosis of their short stature (<3 percentile) and slow height velocity (<−1.0 SD). This study was approved by the Bioethics Committee of the Polish Mother’s Memorial Hospital–Research Institute, Lodz, Poland (approval code no.: 57/2018).

In each child, the body height was assessed with the use of a stadiometer and the height standard deviation score (HSDS) was calculated according to the local centile charts for the child’s age and sex [38]. Height velocity was calculated on the basis of the height gain over a period of at least 6 months of observation per year (in cm/year) according to local standards [38]. Next, the body weight was measured, followed by calculating the body mass index standard deviation score (BMI SDS), according to the same standards [38]. During physical examination, the degree of sexual maturation was recorded, using the Tanner scale.

### 2.2. Data Concerning Perinatal Period, Medical History with Particular Consideration of the Present or Past Gastrointestinal Diseases

The history of patients was analyzed in terms of gestational age, birth weight and birth length, chronic diseases, gastrointestinal (GI) complaints and other endocrinopathies, especially hypothyroidism. In all girls and in boys with visible dysmorphic features, the karyotype assessment was performed. Before admission to the hospital, the current TSH and FT4 levels was measured in each child.

### 2.3. Collecting Data on Endocrine Disorders

During hospitalisation, two stimulation tests for GH secretion were performed. The first one was conducted after clonidine, administered orally (with the dose of 0.15 mg/m^2^ of the body surface), with GH concentration measurements at time 0 and at the 30th, 60th, 90th and 120th minute of the test. The second stimulation test was carried out with use of glucagon in intramuscular administration (in the dose of 30 µg/kg of body weight, not exceeding 1 mg), and with GH concentration measurements at time 0 and at the 90th, 120th, 150th and 180th minute. Based on the results of GHmax values in these tests, GHD was diagnosed if the decreased GH secretion was confirmed (GHmax values < 10 ng/mL).

In each child, the concentration of ghrelin, IGF-1 and leptin was assessed after nocturnal fasting, at 6:00 in the morning, i.e., after approximately 10–12 h from the last meal, which was a supper on the first day of hospitalization. IGF-1 concentrations were calculated as IGF-1 SDS, according to the reference data [39].

Also, the concentration of antibodies against *H. pylori*, was measured in IgA and IgG class, with a simultaneous total concentration of IgA and IgG in serum.

#### Inclusion Criteria for Further Analysis:

On the basis of the obtained data, the following were included into the study group:

(1) height below or equal −2.0 SD (HSDS ≤ −2.0);
(2) height velocity below or equal −1.0 SD (HV SDS ≤ −1.0);
(3) normal GH secretion (more or equal than 10 ng/mL) in stimulation tests (ISS).

#### Exclusion Criteria:

On the basis of the obtained data, the following were excluded from the study group:

(1) children born prematurely or small for gestational age (SGA);
(2) children with chronic diseases, with GI symptoms;
(3) children with genetically determined disorders (Turner, Noonan, Silver-Russell, Prader-Willi, pathogenic variant of SHOX gene);
(4) children with hypothyroidism;
(5) children with GHD.

In conclusion, the research was carried out under the aforementioned research project, the duration of which is planned for two years. We collected data on all the children with short stature who were admitted to our Clinic at that time (*n* = 151). According to exclusion criteria, we excluded from that analysis 23 children with GHD, 13 children with SGA, 6 children with hypothyroidism, 7 children with height position higher than −2.0 SD, 10 children with height velocity higher than −1.0 SD, 8 children with symptoms from GI tract and 7 with genetic abnormalities (2 children with Silver-Russell syndrome, 1 child with Noonan syndrome, 1 child with a pathogenic variant of SHOX gene and 3 girls with Turner syndrome) and 12 children with parents who did not agree for further diagnostics at the Gastroenterology Department.

### 2.4. Collecting Data on Gastrointestinal Tract Diseases

Children whose parents agreed to further proceedings were tested at the Gastroenterology, Allergology and Pediatric Department of PMMH-RI in Lodz. Upper GI endoscopy was performed in each child with mucosal biopsies for urease test and pathology. Other blood, sweat and stool tests were performed, and hydrogen breath tests for detection of oligosymptomatic GI diseases, which might influence the children’s growth rate and development. Patients with diagnosed coeliac disease (*n* = 3), non-specific enteritis (*n* = 1) or cystic fibrosis (*n* = 1) were also excluded from evaluation.

Finally, 61 children (25 girls and 36 boys) with ISS were qualified into the study group, their age varying from 5.03 to 15.24 years (the mean age ± SD: 9.44 ± 3.10 years).

Based on the results of the histological examination and the rapid urease test for the presence of *H. pylori* in the biopsy material taken during the endoscopy of the upper GI tract, patients were divided into the following subgroups: *H. pylori*-positive with confirmed *H. pylori* infection and *H. pylori*-negative with excluded *H. pylori* infection.

Among them, 53 children were prepubertal, while 7 was in II stage of puberty according to the Tanner scale and one boy was in stage 3 of puberty.

### 2.5. Laboratory Analysis

GH concentration was measured using the immunometric method at the Immunohistochemical Laboratory of the Department of Laboratory Diagnostics, PMMH-RI. The measurements were performed with Immulite, DPC assay sets, calibrated according to the WHO IRP 80/505 standard set, with sensitivity: 0.01 ng/mL, range: up to 40 ng/mL, the conversion index: ng/mL × 2.6 = mIU/l, the intra-assay CV: 5.3–6.5% and inter-assay CV: 5.5–6.2%.

IGF-1 was assessed by Immulite, DPC assays, WHO NIBSC 1st IRR 87/518 standard was applied, with the analytical sensitivity of 20 ng/mL, the calibration range up to 1600 ng/mL, intra-assay CV–3.1–4.3% and inter-assay CV–5.8–8.4%.

Total ghrelin was measured by the radioimmunometric assay at the Hormonal Diagnostic Laboratory of Neuroendocrinology Department, Medical University of Lodz. The measurements were performed by Millipore assay sets, with the following sensitivity range: 100–10,000 pg/mL, the intra-assay CV: 3.3–10.0% and inter-assay CV: 14.7–17.8%.

Leptin was measured by ELISA assays at the Hormonal Diagnostic Laboratory of Neuroendocrinology Department, Medical University of Lodz. The measurements were performed by Millipore assay sets, with sensitivity: 0.5 ng/mL, intra-assay CV: 2.6–4.6% and inter-assay CV: 2.6–6.2%, the range of 0.5–100 ng/mL.

### 2.6. Statistical Analysis

Descriptive statistics included the number of patients in particular groups and the values of the analyzed parameters, expressed as the mean ± SD. For comparison between different groups, all age- and sex-dependent variables were expressed as SDS values. The student’s *t*-test was applied when distribution of the variable was normal, while if it was different from normal, a non-parametric statistical test (Mann-Whitney U test) was used for comparisons between groups. Correlations were evaluated using the Pearson’s test. Statistically significant differences were accepted when the *p* value was below 0.05.

## 3. Results

In the studied group of 61 children with ISS, based on the results of urease test and of the histopathological evaluation of the gastric biopsy material, in 15 children (24.6%) the *H. pylori* infection was confirmed (*H. pylori* (+) subgroup), while in 46 children (75.4%) it was excluded (*H. pylori* (−) subgroup). Table 1 presents data referring to the chronological age (CA), height (HSDS), height velocity (HV SDS), body mass (BMI SDS), IGF-1 and maximal values of GH concentrations in both stimulation tests for the *H. pylori* (+) and *H. pylori* (−) subgroups.

The mean chronological age and HSDS were similar in both analyzed subgroups of short children (ns). In addition, the nutrition status parameters did not differ between the *H. pylori* (+) and *H. pylori* (−) subgroups. A significantly lower ghrelin concentration and IGF-1 SDS value was observed in *H. pylori* (+) compared to *H. pylori* (−) children, despite the fact that there were no significant differences in maximal GH concentrations during stimulation tests between the groups. It is to be stressed that the difference between concentrations of ghrelin in the individual groups analyzed on the basis of the student’s *t*-test remained at the border of statistical significance. Therefore, due to the deviation from the normal distribution for ghrelin in the *H. pylori* (+) group (while normal distribution in the *H. pylori* (−) group) and the large dispersion of the standard deviation, the results do not provide certainty as to the reliability of the test result. Nevertheless, the results of post-hoc power calculation analysis confirmed the null thesis. The observed tendency to lower ghrelin levels in children with *H. pylori* (+) seems to be clinically interesting and it deserves further observation. Due to the fact that the concentration of ghrelin physiologically decreases with the age and stage of puberty of children, additional statistical analysis was performed only for 53 prepubertal children (Table 2). A statistically significant result was obtained both in the student’s *t*-test and in the Mann-Whitney U test. In these cases, the computation of the test power also yielded a result confirming the null thesis.

The subsequent stage in the analysis was the evaluation of the correlations between ghrelin and leptin concentration, and individual parameters, analyzed separately for the *H. pylori* (+) and *H. pylori* (−) subgroups of children (Table 3 and Table 4).

In the *H. pylori* (−) subgroup, a statistically significant negative correlation was noticed between the ghrelin concentration in serum and the age of children, while no such correlation was observed in the subgroup of children with *H. pylori* (+).

Similarly, in the evaluation of the correlation between body mass, expressed by BMI SDS values, and ghrelin concentration, a significant negative correlation was identified in the group of children with *H. pylori* (−), while not in the *H. pylori* (+) subgroup.

A significant positive correlation was observed between leptin concentration and the nutritional status of children, expressed by BMI SDS in both analyzed subgroups (children with and without *H. pylori* infection, but we found no differences in serum leptin concentration between both analyzed *H. pylori* (+) and *H. pylori* (−) groups, including after narrowing down the group of children to those who were in the prepubertal period.

**Figure 1:**
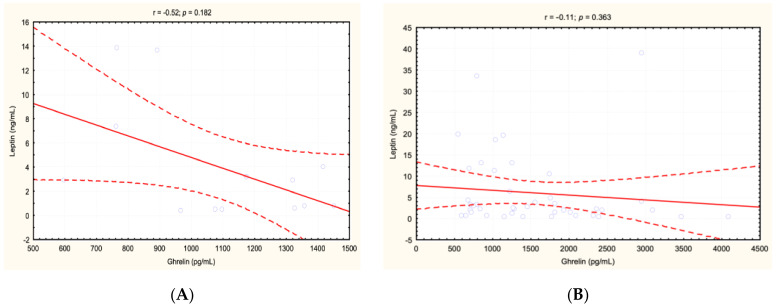
Correlations between ghrelin and leptin concentrations in the group of children with idiopathic short stature and *H. pylori* infection (**A**) and without it (**B**).

No significant correlation was observed between serum ghrelin and leptin concentration in either of the analyzed groups of children (Figure 1).

**Figure 2:**
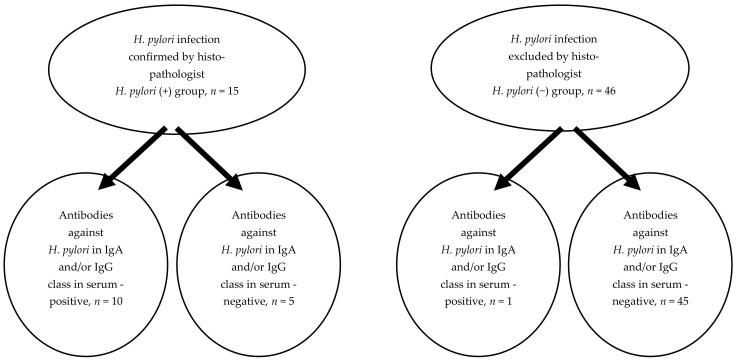
Comparison of the results obtained by the histopathological examination of the biopsy material taken during the endoscopy of upper GI tract with the results of serological test (antibodies against *H. pylori* in IgA and/or IgG class in serum) in analyzed group of ISS children (false negative and false positive results).

It was worth showing that among the 15 children with *H. pylori* (+), an increased concentration of antibodies against *H. pylori* (−) in IgA and/or IgG class was found in only 10 children. Thus, false negative results of serological tests were then obtained in 5 (30%) children. In turn, a false positive result of serological test was noted in one child (*H. pylori* was not confirmed in the histological examination or the rapid urease test biopsy material taken during the endoscopy of the upper GI tract) (Figure 2).

## 4. Discussion

In our study we found that in ISS children with confirmed *H. pylori* infection, both ghrelin and IGF-1 levels are significantly lower than in ISS children without this infection. Additionally, the mean values of the maximum GH concentrations in the stimulation tests were similar in both groups. However, it should be noted that the group we studied was rather small which certainly makes the results less convincing. Our study should be treated as a pilot and the results of statistical analyzes should be repeated on larger groups of children, performed separately on prepubertal children and children in the pubertal period. If in subsequent tests, in children with *H. pylori*, a reduced concentration of ghrelin and an improvement in its concentration are confirmed, and an improvement in the growth rate and IGF-1 concentration after eradication, it may be a reason to include the *H. pylori* infection assessment among the recommended ones in diagnostics ISS. We hope that our research on this unique group of children will contribute to the discussion on this topic and further research in this direction.

**Figure 3:**
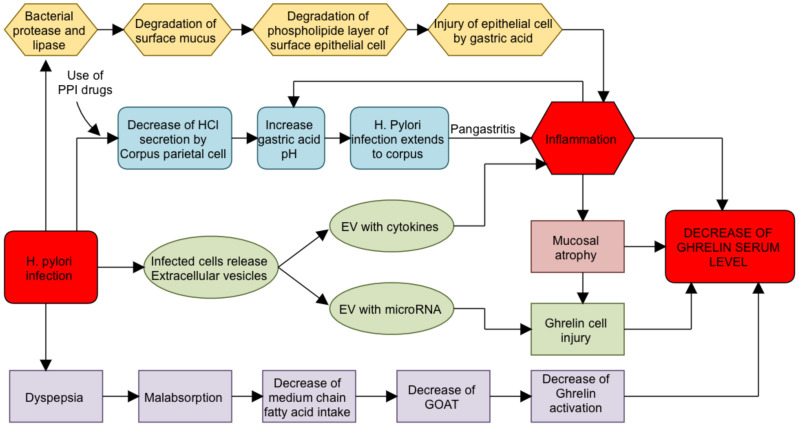
Putative mechanisms that may cause disturbances in ghrelin secretion as a result of *H. pylori* infection. EV—extracellular vesicles; GOAT—ghrelin O-acyltransferase; PPI—proton pump inhibitor.

There are at least three issues that should be discussed. ISS in children is diagnosed when endocrinological and genetic justification cannot be found, GH secretion is normal and there are no coexisting chronic diseases that could interfere with growth processes in any way [1]. However, it is to be expected that this group of children may include patients with still unidentified disorders related to various factors responsible for child development and appetite control [2]. As we mentioned in the introduction, in our previous study, we found out that the prevalence of oligosymptomatic gastrointestinal diseases in children with ISS, which may underlie the growth velocity impairment, is as high as 70% [3]. Among them, *H. pylori* infection was one of the most common problems [3]. However, whether and why *H. pylori* infection affects the growth rate in children is a question raised in this paper. Recently, Xu et al. [9] have published a meta-analysis of 9384 children from 29 studies and found that there is evidence supporting the hypothesis that *H. pylori* infection is associated with growth failure in children [9]. However, what this directly results from is still unclear. The negative effect of the *H. pylori* infection on ghrelin secretion from X/A cells in the gastric oxyntic mucosa is one of possible causes. The putative mechanisms, described in some articles [27,29,40,41], that may cause disturbances in ghrelin secretion as a result of *H. pylori* infection was illustrated by us in Figure 3.

Similar results to ours have been published by several other authors, although their research looked at children without short stature. Yang et al. [6] have shown that ghrelin concentrations are lower in *H. pylori*-positive children and increase after successful eradication with an increasing growth rate. On the other hand, Ozen et al. [15] also observed a lower concentration of ghrelin in children with *H. pylori* infection, although in their observations it was not found to improve the growth of children. It should be noted, however, that the group of children studied by those authors had a diagnosis of *H. pylori* infection based on a serological test and children with *H. pylori* infection were significantly older than those without it. Also, Płonka et al. [42] and Konturek et al. [43] found significantly lower ghrelin levels in children with *H. pylori* infection, although in the latter two studies the effect of eradication on ghrelin secretion was not analyzed and the diagnosis of *H. pylori* infection was based on serological tests.

In turn, Pacifico et al. [19] did not observe any significant differences in ghrelin concentrations in children, either with or without the *H. pylori* infection, although they observed a significant inverse correlation between ghrelin concentration and the histological severity of gastritis. It is worth continuing studies attempting to verify whether the impaired ghrelin secretion is due to the presence of bacteria or inflammation caused by it and whether other gastric inflammations without the *H. pylori* infection may also cause this. In some studies conducted among adults, it has also been demonstrated that significant effects on ghrelin secretion from the stomach are exerted by diseases, concomitant to *H. pylori* infection, such as peptic ulcer, gastric cancer and antral gastritis, or occurring without *H. pylori* infection, such as atrophic gastritis, affecting both stomach fundus and body [44,45,46]. In our material obtained from children without GI clinical symptoms, inflammatory changes were observed in stomach antrum and body, but they were generally limited to mild, lymphocytic, superficial mucositis and in each case, it coexisted with *H. pylori*.

Since *H. pylori* infection reduces ghrelin secretion and, consequently, its concentration in blood serum, it may be assumed that following an effective eradication of the infection, the secretion and concentration of ghrelin should return to normal values, which was confirmed in children and in adults [6,35,36,47]. The duration of the infection, the degree of concomitant inflammatory changes, the influence of the intestinal microflora and the metabolism of the host may have an additional effect on ghrelin production [48,49,50].

Since ghrelin stimulates the secretion of GH from the pituitary gland both directly and indirectly, it is expected that inhibiting its production will reduce the secretion of GH and IGF-1—the main mediator of GH activity responsible for linear growth in children. In our study, we found lower IGF-1 SDS values in children with lower fasting ghrelin serum concentration; however, it was surprising that the GH concentrations in the stimulation tests were comparable in both the *H. pylori* positive and *H. pylori* negative groups. This is further evidence of the limited usefulness of GH stimulation tests, routinely used in the diagnosis of GHD, as they have poor reproducibility and a high false-negative and false-positive rate [51]. Perhaps, for normal growth, the total pool of GH secretion during the day or other mechanisms responsible for this phenomenon are more important. In our previous studies, we proved a positive correlation between nocturnal ghrelin and nocturnal GH secretion [52]. We also found the higher ghrelin concentration in children with neurosecretory dysfunction [53]. It is possible that the spontaneous GH secretion is altered in children with ISS and *H. pylori* infection (e.g., there is an impaired pattern of GH secretion, or a reduced GH peak value, or a reduced mean GH secretion level).

Currently, there is much debate about the treatment of ISS with recombinant human GH (rhGH). Although it brings the expected benefits in terms of improving final height, it seems that the search for the causes of ISS and their subsequent elimination may reduce the costs and psychological burden of such treatment [54,55].

The type of the diagnostic method used plays an important role in detecting *H. pylori*. In many of the studies mentioned above, the diagnosis of *H. pylori* was made on the basis of the presence of anti- *H. pylori* antibodies or the C-urea breath test and only in a few studies on the basis of endoscopic biopsies results, which are currently recommended for the diagnosis of *H. pylori* infection.

In our study, we conducted a gastroscopic examination on all children and, depending on its result, the *H. pylori* infection was confirmed. Owing to this, our diagnosis was reliable, and we were also able to compare the reliability of serological tests. False positive serological test results were obtained in only one child and false negative results in 30% of patients.

Interestingly, the incidence of *H. pylori* in the studied group of children with ISS was similar to that in the general population [26], i.e., 25%. Thus, there is no basis for the claim that *H. pylori* infection can be a common cause of short stature in children. However, in our study, we confirmed that if short stature in a child is accompanied by *H. pylori* infection, the concentrations of ghrelin and IGF-1 are lower than in short stature children and without this infection, especially among prepubertal children. It can be speculated that these disturbances may be the reason for a poorer growth rate in this subgroup. On the other hand, the cause of the short stature in the *H. pylori*-negative subgroup still remains unclear.

The effect of decreased serum ghrelin levels on food intake should also be considered. It has been shown that *H. pylori* infection is able to induce malabsorption of several nutrients, such as iron, cobalamin or vitamins, and patients’ nutritional status improves after eradication therapy [48,50]. It is known that ghrelin stimulates the hunger center and that IGF-1 is reduced in malnourished children. However, in the group of children we analyzed, there were no malnourished children, and both groups had comparable body weight.

We also considered the possible influence of leptin on regulation of appetite. Leptin stimulates anorexigenic centers, reduces appetite and enhances thermogenesis. Since *H. pylori* infection is suspected to increase gastric leptin production (by increasing cytokines during gastric inflammation), it can be expected that these higher levels of leptin (in the children with otherwise normal BMI) will result in poorer appetite (and worse growth velocity) [31,32].

However, our research has not confirmed higher leptin concentration in children with *H. pylori* (+) compared to *H. pylori* (−). We also did not find the correlation between ghrelin and leptin levels significant. It should be emphasized that perhaps the severity and duration of gastritis is important in this aspect, but the children we studied were not analyzed in this way.

## 5. Conclusions

*H. pylori* infection may be responsible for poor ghrelin formation and secretion and, as a further consequence, reduced GH−IGF-1 secretion and worsening of height velocity in children.

Unexplained short stature with a decrease in height velocity in children may be a premise to look for the presence of *H. pylori* infection, especially if in the prepubertal child, a low IGF-1 concentration, despite normal GH results in stimulation tests, is recorded.

## Figures and Tables

**Table 1:** Comparison of growth parameters and hormone tests results in the whole ISS group considering the presence of the *H. pylori* infection.

	*H. pylori (+)*	*H. pylori (−)*	*p =*
*n* (F/M)	15 (6/9)	46 (19/27)	
CA (years)	10.67 ± 3.44	9.85 ± 3.45	0.431
HA (years)	8.16 ± 2.94	7.39 ± 2.80	0.367
HSDS	−2.45 ± 0.63	−2.37 ± 0.93	0.764
HV SDS	−1.55 ± 0.5	−1.25 ± 0.37	0.311
BMI SDS	−0.54 ± 0.65	−0.29 ± 0.93	0.357
ghrelin (pg/mL)	1088.77 ± 271.48	1535.70 ± 838.54	0.048 *
leptin (ng/mL)	4.79	8.58 ± 5.96	0.426
IGF-1 SDS	−1.36 ± 1.36	−0.92 ± 1.07	0.046 *
GH max after clonidine (ng/mL)	17.16 ± 11.69	17.64 ± 10.22	0.498
GH max after glucagon (ng/mL)	8.44 ± 4.71	9.46 ± 5.93	0.395

*H. pylori*—*Helicobacter pylori*, CA—chronological age, HA—height age, HSDS—height standard deviation score, HV SDS—height velocity standard deviation score, BMI SDS—body mass index standard deviation score, GH max—maximal growth hormone concentration during stimulation test, IGF-1 SDS—insulin-like growth factor 1 standard deviation score, *—*p* < 0.05.

**Table 2:** Comparison of growth parameters and hormone tests results of the subgroup of prepubertal children distinguished from the whole analyzed group of ISS children considering the presence of the *H. pylori* infection.

	*H. pylori* (+)	*H. pylori* (−)	*p =*
*n* (F/M)	14 (5/9)	39 (16/23)	
CA (years)	10.25 ± 3.14	8.98 ± 2.87	0.173
HA (years)	7.78 ± 2.65	6.72 ± 2.38	0.173
HSDS	−2.53 ± 0.58	−2.34 ± 0.93	0.486
HV SDS	−1.48 ± 0.31	1.23 ± 0.51	0.341
BMI SDS	−0.58 ± 0.65	0.27 ± 0.92	0.263
ghrelin (pg/mL)	1111.99 ± 265.82	1784.59 ± 1007.15	0.017 *
leptin (ng/mL)	3.14 ± 3.92	6.08 ± 8.99	0.28
IGF-1 SDS	−1.54 ± 1.23	0.91 ± 1.05	0.041 *
GH max after clonidine (ng/mL)	14.53 ± 10.23	16.20 ± 8.91	0.561
GH max after glucagon (ng/mL)	9.69 ± 7.69	10.46 ± 6.30	0.724

*H. pylori—Helicobacter pylori*, CA—chronological age, HA—height age, HSDS—height standard deviation score, HV SDS—height velocity standard deviation score, BMI SDS—body mass index standard deviation score, GH max—maximal growth hormone concentration during stimulation test, IGF-1 SDS—insulin-like growth factor 1 standard deviation score, *—*p* < 0.05.

**Table 3:** Correlations between the analyzed parameters and ghrelin concentration in the group of children with idiopathic short stature depending on the presence of *H. pylori* infection.

	Ghrelin (pg/mL)			
	*H. pylori* (+)		*H. pylori* (−)	
	*r* =	*p* =	*r* =	*p* =
CA (years)	−0.37	0.179	−0.54 *	0
HA (years)	−0.47	0.075	−0.51 *	0
HSDS	−0.53 *	0.043	−0.04	0.638
BMI SDS	−0.14	0.616	−0.43 *	0.006
GH max after clonidine (ng/mL)	−0.14	0.623	0.25	0.152
GH max after glucagon (ng/mL)	0.01	0.963	−0.19	0.265
IGF-1 (ng/mL)	−0.52 *	0.047	−0.50 *	0.001
IGF-1 SDS	−0.25	0.361	−0.17	0.309

*H. pylori—Helicobacter pylori*, CA—chronological age, HA—height age, HSDS—height standard deviation score, BMI SDS—body mass index standard deviation score, GH max—maximal growth hormone concentration during stimulation test, IGF-1—insulin-like growth factor 1, IGF-1 SDS—insulin-like growth factor 1 standard deviation score, *—*p* < 0.05.

**Table 4:** Correlations between the analyzed parameters and leptin concentration in the group of children with idiopathic short stature depending on the presence of *H. pylori* infection.

	Leptin (ng/mL)			
	*H. pylori* (+)		*H. pylori* (−)	
	*r* =	*p* =	*r* =	*p* =
CA (years)	0.47	0.102	0.24	0.14
HA (years)	0.54	0.055	0.26	0.11
HSDS	0.61 *	0.027	−0.01	0.946
BMI SDS	0.66 *	0.015	0.64 *	0
GH max after clonidine (ng/mL)	−0.14	0.333	−0.48 *	0.002
GH max after glucagon (ng/mL)	0.41	0.117	−0.05	0.956
IGF-1 (ng/mL)	0.63 *	0.021	0.38 *	0.016
IGF-1 SDS	0.32	0.282	0.1	0.548

*H. pylori*—*Helicobacter pylori*, CA—chronological age, HA—height age, HSDS—height standard deviation score, BMI SDS—body mass index standard deviation score, GH max—maximal growth hormone concentration during stimulation test, IGF-1—insulin-like growth factor 1, IGF-1 SDS–insulin-like growth factor 1 standard deviation score, *—*p* < 0.05.

## Data Availability

The datasets used and/or analyzed within the framework of this study are available from the corresponding author on reasonable request.
